# Transcriptome annotation and characterization of novel toxins in six scorpion species

**DOI:** 10.1186/s12864-019-6013-6

**Published:** 2019-08-13

**Authors:** Dwin G. B. Grashof, Harald M. I. Kerkkamp, Sandra Afonso, John Archer, D. James Harris, Michael K. Richardson, Freek J. Vonk, Arie van der Meijden

**Affiliations:** 1 IBL, Leiden University, Leiden, The Netherlands; 2 CIBIO/InBio, Vairão, Portugal; 3 Naturalis Biodiversity Center Leiden, Leiden, The Netherlands

**Keywords:** Transcriptome, Scorpion, Venom

## Abstract

**Background:**

Venom has evolved in parallel in multiple animals for the purpose of self-defense, prey capture or both. These venoms typically consist of highly complex mixtures of toxins: diverse bioactive peptides and/or proteins each with a specific pharmacological activity. Because of their specificity, they can be used as experimental tools to study cell mechanisms and develop novel medicines and drugs. It is therefore potentially valuable to explore the venoms of various animals to characterize their toxins and identify novel toxin-families. This study focuses on the annotation and exploration of the transcriptomes of six scorpion species from three different families. The transcriptomes were annotated with a custom-built automated pipeline, primarily consisting of Basic Local Alignment Search Tool searches against UniProt databases and filter steps based on transcript coverage.

**Results:**

We annotated the transcriptomes of four scorpions from the family Buthidae, one from Iuridae and one from Diplocentridae using our annotation pipeline. We found that the four buthid scorpions primarily produce disulfide-bridged ion-channel targeting toxins, while the non-buthid scorpions have a higher abundance of non-disulfide-bridged toxins. Furthermore, analysis of the “unidentified” transcripts resulted in the discovery of six novel putative toxin families containing a total of 37 novel putative toxins. Additionally, 33 novel toxins in existing toxin-families were found. Lastly, 19 novel putative secreted proteins without toxin-like disulfide bonds were found.

**Conclusions:**

We were able to assign most transcripts to a toxin family and classify the venom composition for all six scorpions. In addition to advancing our fundamental knowledge of scorpion venomics, this study may serve as a starting point for future research by facilitating the identification of the venom composition of scorpions and identifying novel putative toxin families.

**Electronic supplementary material:**

The online version of this article (10.1186/s12864-019-6013-6) contains supplementary material, which is available to authorized users.

## Background

Venom has evolved in parallel in multiple animals for self-defense, prey capture or both. Animals that use venom are widely distributed across the tree of life and include snakes, arachnids (including spiders and scorpions), mollusks (including cone snails, octopuses and jellyfish), insects (including bees and beetles) and some teleost fishes (as reviewed in [1]). Venoms are typically complex mixtures of bioactive peptides and/or proteins formally referred to as ‘toxins’. Toxins are very specific in their activity and different toxins may cause very different pharmacological effects. They act by binding to ion-channels and disrupting metabolic pathways. This leads to paralysis, pain, hematological disturbances, immune reactions, necrosis and apoptosis in the animal that has been injected with venom [2, 3]. Because of the specificity of toxins, they can be used as experimental tools or probes to study cell mechanisms and develop novel medicines and drugs [3]. The study of venoms, categorizing the different toxins that constitute a venom and their activities, has already been successful in the development of novel pharmaceuticals, for example the development of the ACE inhibitor Captopril® from the venom of the snake *Bothrops jararaca* [4]. From the venom of the death-stalker scorpion *Leiurus quinquestriatus*, a glioma cell binding toxin is already in use for cancer therapeutics [5]. Other examples are the antimicrobial peptides (AMPs), also found in scorpions, used for treating infections from antibiotic-resistant bacteria, fungi and even viruses [6, 7]. These examples demonstrate the potential benefit of scorpion venom and toxin research in the development of novel medicines. Because of the great diversity, variability, selectivity and application of toxins it is crucial to study additional venoms and especially to identify novel toxins that might be used for the development of new drugs and medicines against for example ion channel-associated diseases like autoimmune diseases, chronic pain, diabetes, epilepsy, and gliomas. However, identifying new toxins for drug development is also challenging since most peptides, like toxins, are easily broken down when ingested or give adverse reactions when injected as drug.

Scorpion venoms typically consist of a complex mixture of small polypeptides, enzymes, nucleotides, lipids, mucoproteins, biogenic amines as well as unidentified substances [8]. In these venom mixtures, polypeptides and enzymes are the most prominent and toxic components [9]. Based on structure and effect, scorpion toxins are generally classified into two classes: disulfide-bridged peptides (DBPs) and non-disulfide-bridged peptides (NDBPs) [9–11]. DBPs have at least two cysteines that interact and form a disulfide bridge. Major scorpion toxin families that have these bridges, in order of medical relevance, are sodium-channel binding toxins (NaTx), potassium-channel binding toxins (KTx), chloride-channel binding toxins (ClTx), calcium-channel binding toxins (CaTx), Kunitz-type toxins and M-theraphotoxins, respectively. These toxin families are also most lethal to humans [9–11]. The other class of toxins, NDBPs, is much more diverse and less studied, both due to their less harmful nature and generally lower levels of expression. There are two sub-groups of NDBPs: cationic and highly acidic peptides [9]. Although some studies have successfully identified multiple highly acidic peptides, these peptides have not yet been functionally categorized [12, 13]. Researchers have recently identified and functionally characterized some of these toxins. Typical biological activities of these NDBPs include antimicrobial, hemolytic, cytolytic and bradykinin-potentiating, making this group extremely diverse ([11], and as reviewed in [14]).

In transcriptome analysis the resolution is often dependent on the amount of data available to annotate the transcripts and published annotated reference genomes that aid the transcript annotation. With only two scorpion genomes currently accessible (*Centruroides sculpturatus* and *Mesobuthus martensii* with 30,465 and 32,016 coding genes, respectively), for which toxin genes were not validated, annotating venom gland transcriptomes becomes inherently difficult [15]. Another major issue for scorpion transcriptomics compared to e.g. snake transcriptomics is the limited availability of genes and proteins used to annotate. The NCBI database holds approximately 30,000 scorpion genes and over 44,000 scorpion proteins, while the same database stores over 114,000 snake genes and 323,000 snake proteins. Furthermore, most of these stored scorpion proteins are housekeeping genes, leaving only 4500 scorpion proteins labelled as scorpion toxin, compared to the 10,000 snake toxins in the NCBI database. This therefore greatly reduces the references that can be used to annotate a scorpion transcriptome, and more specifically scorpion toxin diversity [15]. In addition, the toxin diversity of scorpion venom is in general higher than that of snake venom.

In order to identify new biomedically useful DBP and NDBP toxins, this study has focused on six scorpions belonging to three families. We have included four buthid scorpions: (i) *Androctonus mauritanicus* (ii) *Babycurus gigas* (iii) *Grosphus grandidieri* (iv) *Hottentotta gentili*. Of the 20 scorpion families recognized by Sharma et al. [16] the family Buthidae contains almost all species that are significantly harmful to humans. Approximately 2400 scorpion species have been described, and, of the 30 or so that are considered medically relevant to humans, 29 are from the family Buthidae. This family is known for the abundance of potent ion-channel toxins in its venom. Since buthid scorpions seem the most active pharmacologically, and their venom contains ion-channel targeting toxins, which are medically relevant, the venoms of scorpions from this family have been extensively studied [17]. However, this has diverted attention away from the other scorpion families. Studies have shown that some toxins in non-buthid scorpions possess unique biological activities and applications [18–20]. Therefore we also included one scorpion from the family Iuridae, (v) *Protoiurus kraepelini*, and one scorpion from the family Diplocentridae, (vi) *Nebo hierichonticus*.

The first aim of this study was the identification of the venom composition of the six scorpion species listed above, achieved through high-throughput sequencing and transcriptome analysis. The benefits of using high-throughput sequencing methods are efficiency and speed. Furthermore, this method allows for an easy approach to quantify the coverage of transcripts into expression-related data, and increases the probability of finding novel proteins [17, 21–23]. In this study both the telson (stinger) and the chela (pincer) of each of the six scorpions were sequenced, resulting in two transcriptomes from each scorpion. The chela transcriptomes were then used to filter out any housekeeping transcripts or other general regulatory transcripts from the telson transcriptomes. To study the remaining transcripts of the telson transcriptomes an automated annotation pipeline was used. This pipeline utilizes datasets downloaded from UniProt [24] (downloaded on February 8th 2018) and labeled each transcript as either physiological, toxin and toxin-family or unidentified. With this pipeline, the venom composition of the six scorpions could be categorized. The second aim of this study was to find novel toxins or novel toxin families. This was done by selecting highly expressed unidentified transcripts from the transcriptomes. For these transcript typical toxin-like features were predicted if present, like a signal peptides (an essential structure of each scorpion toxin), cystine pattern and other conserved domains.

**Fig. 1:**
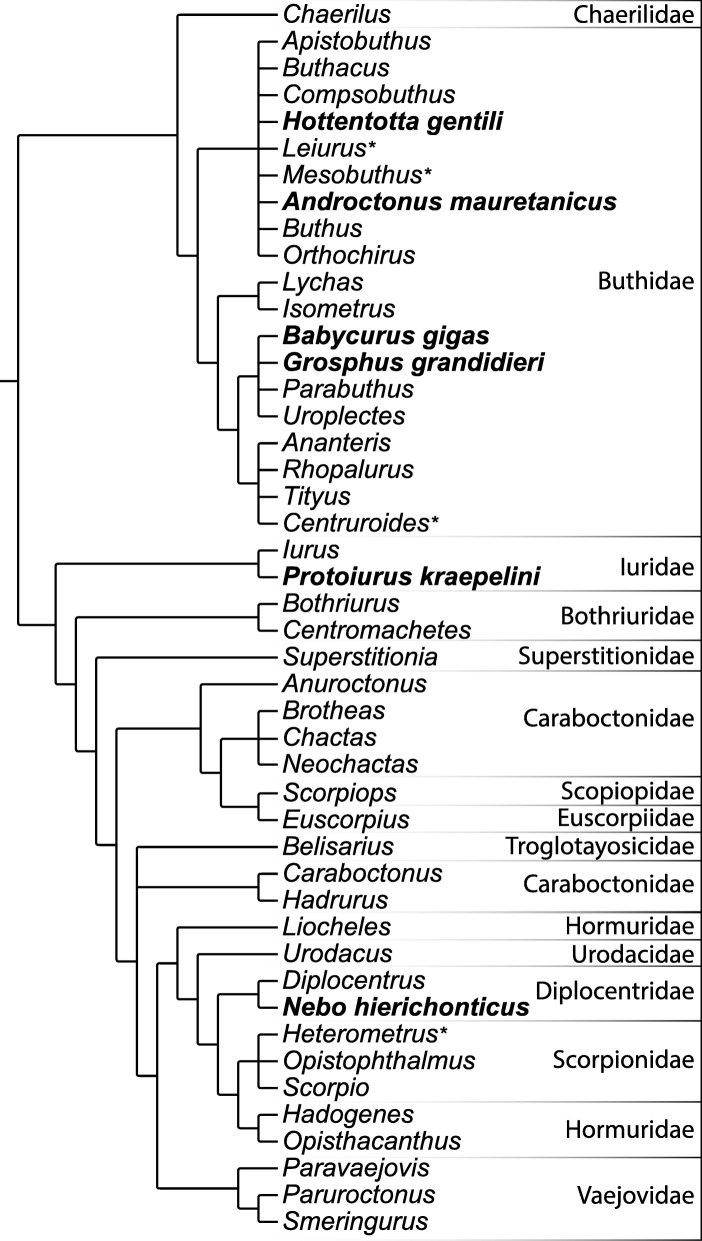
Phylogenetic position of the species used in this study, indicated in bold font. Other species mentioned in the manuscript are indicated with an asterisk. Phylogeny and taxonomy largely based on Sharma et al. [16] and Santibáñez-López [34]. Some taxa, such as *Nebo hierichonticus*, were placed in the tree based only on taxonomic affiliation

This study is, to our knowledge, the first that has focused on the transcriptomics of multiple scorpion families (Fig. 1). The high-throughput sequencing approach increased the probability of finding novel toxins and provided enough data for comparative transcriptomics. From both Iuridae and Diplocentridae no transcriptomic studies have previously been conducted and no venom studies have been conducted for Diplocentridae. We expect that this wide taxonomic approach will increase the chances of identifying novel peptides.

## Methods

Tissue samples of one specimen for each of six species were obtained from captive scorpions (see Additional file 1: Table S3). Specimens were milked by electrostimulation (a square wave with an amplitude of 18 V and a frequency of 45 Hz applied between the 2nd and 5th metasomal segment) five days prior to being sacrificed to ensure the active transcription of venom genes. The scorpions were anesthetized using isoflurane, and subsequently frozen in liquid nitrogen. The telson and chelae were removed and stored separately at − 80 °C until library preparation. RNA extractions were performed using the RNeasy Mini Kit (Qiagen) with quality tested by doing a RIN test using a Tapestation 2200. Library prep was done using the TruSeq RNA Library Prep Kit v2 (Illumina). Sequencing of the RNA samples was done on an Illumina Hiseq 1500. All samples were sequenced with a unique index sequence with read lengths of ~ 280 bp. Sequences were pair-ended (2 × 125 bp). The chela and telson were sequenced separately for each scorpion species, resulting in two transcriptomes per scorpion and 12 transcriptomes in total. Reads were quality filtered using Trimmomatic version 0.36 [25]. The leading 3 and trailing 10 bases were removed from each read. A sliding window of length 4 was used with a quality threshold of 15. Reads less than 50 base pairs were removed. De novo assembly was then performed using Trinity version 2.0.3 using standard parameters [26]. Following assembly reads were clustered using CD-HIT version 4.6.6 with a clustering threshold of 0.95 [27, 28]. The outputs from CD-HIT were our final assembled transcriptomes.

In order to annotate the scorpion transcriptomes two databases were downloaded from UniProt [24] and then merged into one annotated database. The first database was downloaded by filtering for arthropod proteins using the “advanced search option” followed by downloading only the “reviewed arthropod proteins” to increase accuracy and relevance during annotation. The second dataset was downloaded by again selecting the “reviewed arthropod proteins”, and then expanding the filtering by adding the extra option: “Expression -> Tissue specificity -> Toxin or Venom -> Any assertion method”. This resulted in an arthropod dataset containing 12,291 proteins and a subset consisting of arthropod venom and toxins containing 2737 proteins. These datasets were downloaded on the 8th of February, 2018. The second dataset was then annotated manually with the Basic Local Alignment Tool (BLAST, as implemented in the standalone executable, version 2.7.1), protein search (BLASTp) [29], using one to four representatives from major scorpion families found in literature [30]. The representatives were chosen by searching for reviewed proteins for each major scorpion venom in UniProt. The BLASTp was set with a maximum e-value of 1e^− 1^. The toxin and venom proteins were labeled according to their highest similarity with the representatives from the major scorpion toxin families. Toxin or venom proteins in the dataset with no similarity to the representatives from the major scorpion toxin families were labeled as “other toxins”. Using this strategy 1589 proteins of the 2737 proteins could be assigned to one of the major scorpion toxin-families. The remaining 1148 toxins were labeled as “other toxins”. The last step of the database construction was to merge the first dataset and the annotated second dataset based on protein ID. This ensured that all toxins in the first dataset were labeled as either a member of a toxin-family or as “other toxin”. The remaining proteins in the database were then labeled as “physiological”.

To annotate the telson transcriptomes a custom pipeline was constructed, largely in BioPython (version 1.70), a module for Python (version 3.6.4). This custom bioinformatics pipeline follows seven steps leading to full annotation of the transcriptomes: (i) The pipeline calculates the coverage by: average read length (150) * read count of the transcript / length of the transcript. With this formula the coverage of a single transcript is normalized by its size, making the transcripts comparable to each other. (ii) The pipeline removes highly similar transcripts that are expressed in common between the telson and chela transcriptomes of the same species by performing a BLASTn with the following parameters: e-value = 1e^− 50^; output format = 6; max target sequences = 1; minimal percentage identity = 99%; minimal percentage coverage = 95%. This removes most housekeeping transcripts from the telson transcriptome. Since no venom or toxin genes are likely to be expressed in the chela, all toxin and venom transcripts, together with some physiological transcripts not expressed in the chela, are kept in the transcriptome. (iii) For every transcript left in the transcriptome an open reading frame (ORF) is predicted with the ORFFinder algorithm on NCBI’s web portal to increase speed, accuracy and relevance of the next steps. This step also removes many partial and incomplete transcripts. (iv) The fourth step is the actual annotation using BLASTp and the previously created annotated database filled with both physiological and toxin arthropod proteins. The ORF of every transcript is blasted against the annotated database, using the parameters: e-value 1e^− 5^; output format = 6; max subject sequences = 1. The transcripts are then labeled based on the label of their BLASTp hit or considered “unidentified”. (v) Transcripts with a calculated coverage value lower that 5 are then removed, since those transcripts have a higher chance of being misassembled and are assumed to be insignificant in the venom of the scorpion. (vi) Then all transcripts that are found to have an orthologue in the chela transcriptome are labeled as “physiological”, and the remaining transcripts are labeled according to the label of their BLASTp hit to assign them to toxin-families. All transcripts labeled as a member of a toxin family or labeled as “other toxin” are considered part of the venom. (vii) Lastly, for highly expressed toxins a signal peptide is predicted as validation of the annotation, by uploading the transcript’s ORF to SignalP with the SignalP sensitivity set on “Sensitive” [31].

To find novel toxins, the focus was on the big groups of “unidentified” transcripts, since those transcripts could not be annotated by our annotated database. To increase relevance the “unidentified” transcripts with a relative expression of at least 0.5% of the total transcriptome expression were considered “high expressed unidentified”. Of those “high expressed unidentified” ORFs were predicted with the ORFFinder algorithm on NCBI’s web portal (parameters: ORF start codon to use = “ATG only”) in all frames and then signal peptides were predicted (parameters: D-cutoff values = “Sensitive”) [24, 27]. The longest ORF with the highest SignalP score was selected as the correct ORF for a transcript. Transcripts were then grouped based on their signal peptide, C bridges and conserved domains. To expand these groups with transcripts with a lower expression, or to identify these groups, the “high expressed unidentified” were BLASTed locally against the pooled telson transcriptomes of the six scorpions (parameters: e-value 1; output format = 6; max subject sequences = 1). Of all the hits found for a “high expressed unidentified” transcript, their signal peptide was predicted. If a hit did not have either a signal peptide or a coverage above 5 it was discarded; otherwise it was added to the same cluster that the “high expressed unidentified” transcript belonged to. Both the “high expressed unidentified” and their hits were BLASTed against the complete non-redundant protein database of NCBI in order to identify the groups or label them as novel. The different groups were then aligned using the alignment software of CLC Main Workbench 7 (parameters: gap open = 10.0; Gap extension cost = 1.0; End gap cost = As any other; Alignment = Very accurate). If members of the group had signal peptides, conserved C bridges or other conserved domains, and they could not be identified using the non-redundant database, they were labeled as a putative novel toxin-family.

## Results

Assembly of the chela and telson transcriptomes of the six scorpion species resulted in comparable transcript numbers, both before and after the coverage cutoff set at 5 (Table 1). The telson transcriptomes were run through the bioinformatics pipeline, and each transcript labeled as “toxin”, “physiological” or “unidentified”. The coverage cutoff and chela orthologue cutoff resulted in divergent numbers of transcripts across the six transcriptomes (Table 2). The toxin expression levels ranged from 8477 in *B. gigas* to 26,005 in *H. gentili*. However, the toxin expression levels in the groups “physiological” and “toxin” were more similar, ranging from 3250 in *B. gigas* to 5178 in *H. gentili* and from 79 in *N. hierichonticus* to 317 in *H. gentili* for the “physiological” and “toxin” groups, respectively. Most of the differences therefore were due to the “unidentified” group of transcripts.

**Table 1:** Assembly statistics and coverage cutoff statistics of the chela and telson transcriptomes per scorpion species

	NCBI accession number	# of transcripts	Average coverage	# of transcripts after coverage cutoff	Average coverage after coverage cutoff
*A. mauritanicus* (telson)	SAMN12385121	92,307	33.5	28,563 (30%)	103.4
*A. maurtanicus* (chela)	SAMN12385122	66,949	25.5	20,970 (31%)	76.6
*B. gigas* (telson)	SAMN12385123	49,557	66.5	17,357 (35%)	185.8
*B. gigas* (chela)	SAMN12385124	65,083	29.7	19,125 (30%)	96.2
*G. grandidieri* (telson)	SAMN12385125	58,014	66.5	18,414 (32%)	205.0
*G. grandidieri* (chela)	SAMN12385126	46,313	39.0	15,863 (34%)	109.8
*H. gentili* (telson)	SAMN12385127	70,182	95.8	37,545 (54%)	176.2
*H. gentili* (chela)	SAMN12385128	50,776	69.7	28,630 (56%)	121.1
*P. kraepelini* (telson)	SAMN12385129	64,672	97.1	35,220 (54%)	175.6
*P. kraepelini* (chela)	SAMN12385130	46,614	74.3	25,872 (56%)	131.2
*N. hierichonticus* (telson)	SAMN12385131	68,269	29.0	21,201 (31%)	88.6
*N. hierichonticus* (chela)	SAMN12385132	55,549	30.9	16,621 (30%)	98.2

**Table 2:** Toxin expression levels in the telson transcriptomes of the six scorpion species after the coverage cutoff of 5 and the orthologue cutoff, together with the expression levels of transcripts labelled as “physiological”, “toxin” or “unidentified” by the bioinformatics pipeline described in the method section

	# of transcripts after cutoffs	# of “physiological” labelled transcripts	# of “toxin” labelled transcripts	# of “unidentified” labelled transcripts
*A. mauritanicus*	20,048	5100	247	14,701
*B. gigas*	8477	3240	134	5103
*G. grandidieri*	10,937	3733	179	7025
*H. gentili*	26,005	5178	317	20,510
*P. kraepelini*	22,848	4516	130	18,202
*N. hierichonticus*	12,857	3750	79	9028

**Fig. 2:**
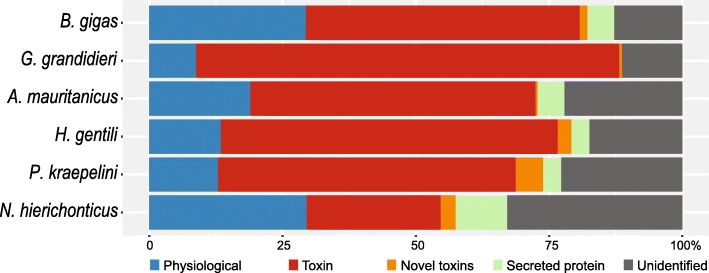
Coverage of the transcripts in the transcriptomes grouped by their label, per scorpion species. The coverage is an indicator of the expression in the telson tissue

The coverage data of a transcript can be used as a very rough indicator of the expression of that transcript in the transcriptome. The transcripts were grouped on label to show the relative expression of each of the labels in the transcriptome per scorpion species, resulting in a percentage of the total transcript number of the entire transcriptome (Fig. 2). However, since only single individuals were sequenced per species, and expression not standardized against housekeeping genes, these numbers should be interpreted with caution. The buthid scorpions and *P. kraepelini* have similar toxin expression, namely between 51 and 56%, with *G. grandidieri* at the top with 74%. All four buthid scorpions had more ion channel targeting toxins, based on raw transcript count, than non-buthids (Table 3). The non-buthids *P. kraepelini* and *N. hierichonticus* both appear to have low numbers of ion-channel targeting toxins and more NDBP toxins. In particular, *P. kraepelini* has the highest abundance of bradykinin potentiating peptide-like transcripts and most phospholipase A2-like transcripts compared to the other investigated scorpions.

**Table 3:** Composition of the transcripts labelled as toxin, shown per scorpion species

	*A. mauritanicus*	*B. gigas*	*G. grandidieri*	*H. gentili*	*P. kraepelini*	*N. hierichonticus*
α-NaTx	25	9	8	25	2	1
β-NaTx	17	4	14	27	4	4
α-KTX	20	11	8	24	2	2
β-KTX	5	2	2	8	3	3
γ-KTX	10	7	5	19	2	2
κ-KTX	0	0	0	1	1	1
Clorotoxin	2	2	4	7	2	2
CaTx	2	3	0	7	2	0
Kunitz-type	9	11	3	15	6	1
M-theraphotoxin	3	6	4	4	1	3
Bradykinin potentiating peptide (BPP)	3	1	1	6	10	8
BmKa2-like	30	6	14	33	3	1
Phospholipase A2 (PLA2)	10	7	3	6	18	5
Other toxins	111	65	113	135	74	46
Total toxins	247	134	179	317	130	79

**Fig. 3:**
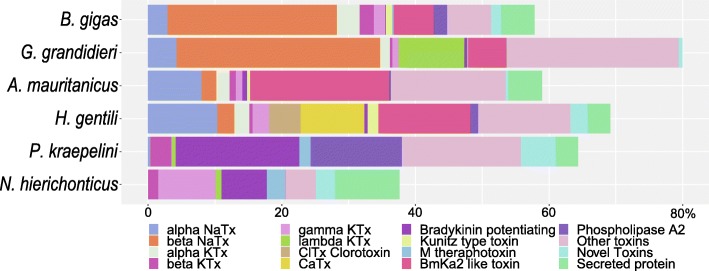
Toxin-family coverage based expression of six scorpion species. The coverage is shown as a percentage of the total expression for that scorpion

Expression levels across toxin families (Fig. 3) show notable differences when compared to transcript count numbers (Table 3). In particular, although “other toxins” made up the largest group by transcript count, each toxin family within this group was relatively less expressed than the major toxin families. Although *A. mauritanicus* and *H. gentili* had most β-NaTx labeled toxins, the expression of the β-NaTx family in *B. gigas* and *G. grandidieri* was higher. Especially for *B. gigas*, where only four β-NaTx toxins were found, that toxin-family had an expression of 25% of the whole transcriptome expression. However, since only single individuals were sequenced, and sequencing was not repeated, all expression data in this study should be viewed with caution.

Within the unidentified transcripts, our approach, as detailed in the methods section, distinguished 53 transcripts as “highly expressed” of which 39 had a signal peptide (Additional file 1: Table S1). These 39 transcripts were clustered based on signal peptide, C-pattern and conserved residues resulting in in five clusters and 19 singlets. Expanding these clusters with a BLASTp search against the pooled telson transcriptomes resulted in 15 clusters and nine singlets, respectively (Additional file 1: Table S2). Of these, nine clusters and two singlets appeared to have toxin-like structures indicating that the 64 transcripts in these clusters and singlets have a higher chance of being a toxin. The last identification step of the assessed “unidentified” clusters, BLASTp searches against the non-redundant database of NCBI, resulted in six novel putative toxin families and two novel putative toxins, with a total of 37 novel putative toxins. Furthermore, we found 33 novel toxins in existing toxin-families like the lambda-potassium channel toxin-family, buthitoxin toxin-family and a neuropeptide toxin-family. Lastly, 19 novel putative secreted proteins without toxin-like disulfide bonds were found (Table 4 and Additional file).

**Table 4:** Additional information about the identification of the clusters done with BLASTp searches against NCBI’s non-redundant database

Cluster name	Best BLASTp hit	Signal peptide	C pattern	Conserved residues	New cluster/singlet label
Cluster 1	Lamda-potassium channel toxin (ADT64271.1)	Some	Yes^a^	High	New toxins in the lamda-potassium channel toxin-family
Cluster 2	Hypothetical secreted protein (ADY39531.1)	High	Yes^a^	High	Novel putative toxin-family 1
Cluster 3	U6-buthitoxin-Hj1a (ADY39519.1)	High	Yes^a^	High	New toxins in the buthitoxin family
Cluster 4	Orphan peptide AbOp-11 (AIX87714.1)	High	N.A.	High	Novel putative secreted proteins
Cluster 5^a^	Hypothetical secreted protein (ADY39514.1)	High	Yes^a^	High	Novel putative toxin-family 2
Cluster 6	venom peptide HtC4Tx1(AOF40173.1)	Low	Yes^a^	Low	Novel putative toxin-family 3
Cluster 7	hypothetical protein (WP_063562212.1)	Low	No^a^	Low	Novel putative toxin-family 4
Cluster 8	Orphan peptide AbOp-18 (AIX87708.1)	High	N.A.	High	New toxins in the neuropeptide toxin-families
Cluster 9	Venom toxin meuTx23 (AMX81480.1)	Low	N.A.	High	New toxins related to meuTx23
Cluster 10	Hypothetical secreted protein (ADY39511.1)	High	N.A.	Low	Novel putative secreted proteins
Cluster 11	RNA-binding protein, putative (SCO66159.1)	Low	N.A.	Some	Novel putative secreted proteins
Cluster 12	Uncharacterized protein (XP_023221782.1)	High	Yes^a^	High	Novel putative toxin-family 5
Cluster 13	Potassium channel toxin alpha-KTx 4.5 (Q5G8B6.1)	Low	Yes^a^	High	New toxins in the potassium channel toxin alpha-KTx 4.5 toxin family
Cluster 14	Hypothetical protein (AEX09189.1)	High	N.A.	Some	Novel putative secreted proteins
Cluster 15	Hypothetical protein (GAU10035.1)	Low	Yes^a^	Some	Novel putative short toxin family 6
Singlet 1	No Hit	N.A	N.A	N.A	Novel putative secreted protein
Singlet 2	No Hit	N.A.	N.A.	N.A.	Novel putative secreted protein
Singlet 3	Hypothetical protein (AEX09189.1)	High	N.A.	Some	Novel putative secreted protein
Singlet 4	Orphan peptide AbOp-11 (AIX87714.1)	High	N.A.	Low	Novel putative secreted protein
Singlet 5	Potassium channel toxin kappa-KTx (P0DJ41.1)	Some	Yes^a^	Low	New potassium channel toxin
Singlet 6	Venom peptide Htgkr2 (AOF40260.1)	Some	N.A.	Low	Novel putative secreted protein
Singlet 7	No Hit	N.A	N.A^a^	N.A	Novel putative toxin 1
Singlet 8	SH3 domain and tetratricopeptide repeat-containing protein (XP_004574858.2)	Low	N.A.	Low	Novel putative secreted protein
Singlet 9	Putative antimicrobial peptide (AEX09192.1)	High	N.A.	High	Novel putative AMP

^a^Indicates clusters or singlets with a conserved C pattern

## Discussion

The species with the lowest toxin expression is *N. hierichonticus*, with a relative toxin expression of 23%. This could be because *N. hierichonticus* is the least dangerous scorpion of the six, suggesting that its venom production is less abundant. However, it is also noteworthy that *N. hierichonticus* has most expression of transcripts labeled as “unidentified”. This is congruent with the fact that it is one of the scorpions from an understudied scorpion family. The scorpion *B. gigas* has about as much relative expression of “physiological” transcripts as *N. hierichonticus*, but without the high expression in “unidentified” transcripts. This might indicate that the visibly low expression of toxins is more due to labeling the toxins as “unidentified”, rather than this scorpion actually producing fewer toxins. The percentages of expression relative to the total that we report allow only a rough comparison of expression levels between the species, and should be viewed with caution. Calculating toxin expression relative to the expression of “housekeeping genes” should allow for a more direct comparisons of expression of toxin genes between the scorpions.

The toxin-families found in *P. kraepelini* in this study are congruent with other studies which also found both ion-channel targeting toxins and enzymatic NDBP toxins [32].

All four buthid scorpions having more ion channel targeting toxins, based on raw transcript count, than non-buthids. This pattern could exist because of the high abundance of ion-channel targeting toxins in the annotated database, due to the extensive studies on ion-channel targeting toxins in Buthidae. Both *A. mauritanicus* and *H. gentili*, the two more medically relevant species in our panel, had striking numbers of ion-channel targeting toxins, and their toxin-family distribution was also quite similar. The relative closely related *Babycurus gigas* and *G. grandidieri* also shared some similarities. However, these did have some key differences in the β-NaTx family, where 10 more β-NaTx-like transcripts were found in the transcriptome of *G. grandidieri*. On the other hand, the toxin families CaTx and Kunitz-type were more abundant in the transcriptome of *B. gigas*. It is important to note that all six scorpions had more transcripts in the group labelled “other toxin”. This shows that the annotated database requires further annotation to obtain a more complete picture of the venom composition. One problem regarding this is the inconsistency of scorpion toxin nomenclature in the literature. A viable solution to this issue is to use both reviewed UniProt labels as well as toxin similarity as a backbone for the annotation of the database.

It is interesting that *H. gentili* was the only buthid scorpion with a κ-KTX labelled transcript, although the transcript had a low expression. This would be the first κ-KTX toxin found in buthid scorpions according to [22]. To confirm this label, the κ-KTX labelled transcript was BLAST-searched against the non-redundant database of NCBI [33]. This showed high similarity with the already published κ-KTX toxin HSP009C (NCBI accession: P0DJ33) in *Heterometrus petersii,* of the family Scorpionidae (one amino acid polymorphism in 62 amino acids). This indicates that *H. gentili* does have κ-KTX type toxins. It is unknown if this is the only buthid scorpion with κ-KTX type toxins; closely related scorpions of the genus *Hottentotta* should be assessed to see whether this is genus-specific.

With the exception of *G. grandidieri*, the buthid scorpions had a significant expression of BmKa2-like toxins. This level of expression is four to 10 times higher than previously reported for buthids [9]. Even though some ion channel targeting toxins were found in the transcriptome of both *P. kraepelini* and *N. hierichonticus*, the expression of those toxins is lower than that of the NDBP toxins.

## Conclusions

By using our custom annotation pipeline it was possible to annotate most transcripts and describe the venom composition in all six scorpions. As expected, the venom compositions had similarities with those that have been published previously, which indicates the accuracy of the pipeline. High-throughput sequencing, and sequencing both the telson and chela allowed for coverage calculation and orthologue filtering, respectively, which were both crucial for finding novel putative toxin-families. The methods for identifying novel putative toxin families appear to be successful. Six novel putative toxin families and two novel putative toxins were found, with a total of 37 novel putative toxins. Furthermore, 33 novel toxins in existing toxin-families, such as the λ-potassium channel toxin-family, buthitoxin toxin-family and a neuropeptide toxin-family were identified. Finally, 19 novel putative secreted proteins were found. Future work should include functional studies and proteomics of the novel putative toxin candidates. With this study a base has been generated for future research of scorpion venomics in the identification of novel putative toxin families. In particular the importance of assessing venoms from different lineages of scorpions was demonstrated by the unique compositions identified in the non-buthids.

## Additional file

Additional file 1: **Table S1.** Number of “highly expressed unidentified” (“unidentified” transcripts with a percentage coverage of 0.5% compared with the total transcriptome coverage) with and without signal peptides. **Table S2.** Number of transcripts in each of the “high expressed unidentified” clusters between each of the steps described in the Methods section. The last column shows the first letter of the species names that are represented by transcripts in that cluster (A = *A. mauretanicus*, B = *B. gigas*, G = *G. grandidieri*, H = *H. gentili*, P = *P. kraepelini,* N *= N. hierichonticus*, All = transcripts of all six species are found in that cluster). **Table S3.** Origin of the samples with the coordinates of capture where available. (DOCX 29 kb)

## Data Availability

The data have been deposited with links to BioProject accession number PRJNA556947 in the NCBI BioProject database (https://www.ncbi.nlm.nih.gov/bioproject/).
