# Supplementary material for: Transcriptome annotation and characterization of novel toxins in six scorpion species
Source: BMC Genomics. 2019 Aug 13;20:645. doi: 10.1186/s12864-019-6013-6 (PMC6693263; doi:10.1186/s12864-019-6013-6)
Supplement: Supplementary file 1 — Table S1. Number of “highly expressed unidentified” (“unidentified” transcripts with a percentage coverage of 0.5% compared with the total transcriptome coverage) with and without signal peptides. Table S2. Number of transcripts in each of the “high expressed unidentified” clusters between each of the steps described in the Methods section. The last column shows the first letter of the species names that are represented by transcripts in that cluster (A = A. mauretanicus, B = B. gigas, G = G. grandidieri, H = H. gentili, P = P. kraepelini, N = N. hierichonticus, All = transcripts of all six species are found in that cluster). Table S3. Origin of the samples with the coordinates of capture where available. (DOCX 29 kb) [file 12864_2019_6013_MOESM1_ESM.docx]

**Supplementary Material**

Supplementary table 1: Number of “highly expressed unidentified” (“unidentified” transcripts with a percentage coverage of 0.5% compared with the total transcriptome coverage) with and without signal peptides.

|  | **# of “high expressed unidentified”** | | **# with signal peptide** |
| --- | --- | --- | --- |
| *Androctonus mauretanicus* | | 7 | 5 |
| *Babycurus gigas* | | 9 | 6 |
| *Grosphus grandidieri* | | 5 | 3 |
| *Hottentotta gentili* | | 10 | 9 |
| *Protoiurus kraepelini* | | 11 | 9 |
| *Nebo hierichonticus* | | 11 | 7 |

**Supplementary table 2:** Number of transcripts in each of the “high expressed unidentified” clusters between each of the steps described in the Methods section. The last column shows the first letter of the species names that are represented by transcripts in that cluster (A = *A. mauretanicus*, B= *B. gigas*, G = *G. grandidieri*, H = *H. gentili*, P = *P. kraepelini,* N *= N. hierichonticus*, All = transcripts of all six species are found in that cluster).

| **Cluster name** | **# of “high expressed unidentified”** | **# of transcripts in the group after BLAST** | **# with signal peptide** | **# with coverage higher than 5** | **# after alignment** | **Present in which scorpion species** |
| --- | --- | --- | --- | --- | --- | --- |
| [Cluster 1](#Cluster_1_Novel) | 2 | 38 | 26 | 23 | 14 | B, G, H |
| [Cluster 2](#Cluster_2_Novel) | 1 | 34 | 25 | 22 | 18 | All |
| [Cluster 3](#Cluster_3_novel) | 5 | 21 | 16 | 16 | 10 | A, H |
| [Cluster 4](#Cluster_4_Novel) | 7 | 28 | 13 | 11 | 7 | A, B, G, H |
| [Cluster 5](#Cluster_5_Novel) | 3 | 16 | 10 | 9 | 7 | All |
| Cluster 6 | 3 | 11 | 5 | 5 | 5 | P, N |
| Cluster 7 | 1 | 6 | 5 | 5 | 2 | P |
| Cluster 8 | 1 | 7 | 5 | 5 | 2 | A |
| Cluster 9 | 1 | 7 | 5 | 4 | 3 | G, H |
| [Cluster 10](#Cluster_11_Novel) | 1 | 4 | 3 | 3 | 2 | A |
| [Cluster 11](#Cluster_12_Novel) | 1 | 8 | 3 | 3 | 2 | A, H |
| [Cluster 12](#Cluster_13_Novel) | 1 | 8 | 4 | 2 | 2 | H, N |
| Cluster 13 | 1 | 3 | 3 | 2 | 2 | B |
| Cluster 14 | 1 | 2 | 2 | 2 | 2 | N |
| Cluster 15 | 1 | 2 | 2 | 2 | 2 | P |
| [Singlet 1](#Singlet_1_Novel) | 1 | 3 | 2 | 2 | 1 | B |
| [Singlet 2](#Singlet_2_Novel) | 1 | 2 | 2 | 1 | 1 | B |
| [Singlet 3](#Singlet_3_Novel) | 1 | 1 | 1 | 1 | 1 | P |
| [Singlet 4](#Singlet_4_Novel) | 1 | 2 | 1 | 1 | 1 | H |
| [Singlet 5](#Singlet_5_Novel) | 1 | 1 | 1 | 1 | 1 | P |
| [Singlet 6](#Singlet_6_Novel) | 1 | 7 | 6 | 6 | 1 | N |
| [Singlet 7](#Singlet_7_Novel) | 1 | 7 | 3 | 2 | 1 | P |
| [Singlet 8](#Singlet_8_Novel) | 1 | 2 | 2 | 2 | 1 | P |
| [Singlet 9](#Singlet_9_Novel) | 1 | 1 | 1 | 1 | 1 | N |

**Footnotes to Supplementary Table 2**

#### Cluster 1: Novel lamda-potassium channel toxins

The best BLASTp hit of cluster one indicated that cluster member “Gros_TR25479 c0_g1_i1” was related to the lamda-potassium channel toxin (ADT64271.1) from *Mesobuthus eupeus*. They had a similar C-pattern, conserved residues and some similarities in the signal peptide as well. Therefore, all members in cluster one are identified as new toxins in the lamda-potassium channel toxin-family.

#### Cluster 2: Novel putative toxin-family 1

BLASTp searches show that closely related toxins have previously been reported in several species in the Buthidae family. The best hit of this cluster is between cluster member “Hot_TR10976 c1_g1_i1” and hypothetical secreted protein (ADY39531.1) from *Hottentotta judaicus*. Since this toxin has not been found in non-buthid scorpions and since the hits were not previously linked to toxins, this cluster is labelled as a ‘novel putative toxin-family’. Similarity is based on signal peptide and conserved C-pattern. Since all six scorpions are represented in this cluster it is possible that this is a scorpion wide family.

#### Cluster 3: novel buthitoxins

The best BLASTp hits of this cluster was between cluster member “Hot_TR15938_c2_g1_i2” and U6-buthitoxin-Hj1a (ADY39519.1) from *Hottentotta judaicus*. Very high similarity suggests that these are closely related. Therefore, this cluster was identified as members of the same buthitoxin toxin-family. Buthitoxins were not previously found in species outside the *Hottentotta* genus. However, this study presents a buthitoxin found in *A. mauretanicus.*

#### Cluster 4: Novel putative secreted proteins

The best BLASTp hit was between “Andy_TR3518_c0_g2_i1” and orphan peptide AbOp-11 (AIX87714.1) from *Androctonus bicolor*. This cluster was labelled as a novel putative secreted-protein-family. Closely related proteins have not been found apart from the blast hit noted above. Furthermore, closely related cluster members have been found in all four Buthidae scorpions and not in any non-Buthidae scorpions. Therefore, it is possible that this is a buthid-specific toxin-family. It is labeled as novel putative secreted-proteins because of the high expression and signal peptide but the lack of a C-pattern. Functional studies in the future could determine whether these transcripts are toxins.

#### Cluster 5: Novel putative toxin-family 2

For cluster five the best BLASTp hit was between “Hot_TR9673_c0_g1_i1” and hypothetical secreted protein (ADY39514.1) from *Hottentotta judaicus*. This cluster is labelled as a novel putative toxin-family since it has a typical conserved C pattern, high telson specific expression and a signal peptide. Furthermore, related BLASTp hits were not previously labelled as a toxin. Since all six scorpions are represented in this cluster, this novel putative toxin-family may be widely represented across the scorpion phylogeny.

#### Cluster 6: Novel putative toxin-family 3

For cluster five the best BLASTp hit was between “Nebo_TR1242_c0_g2_i1” and venom peptide HtC4Tx1(AOF40173.1). This cluster was labelled as a novel putative toxin-family since it has a typical conserved C pattern, high telson specific expression, a signal peptide and could not be identified by its BLAST search. Furthermore, the best BLASTp hit had only a conserved C-pattern but no conserved signal peptide and low similarity in conserved residues. Therefore, the hit is not considered to be a part of this novel putative toxin-family.

#### Cluster 7: Novel putative toxin-family 4

The best BLASTp hit of this cluster was between “Iurus_TR13207_c0_g1_i1” and a hypothetical protein (WP_063562212.1) from *Bacillus horikoshii*. This cluster was labelled as a novel putative toxin-family since it has a typical conserved C pattern, high telson specific expression and a signal peptide. The best BLASTp hit did not have the same C-pattern, and only a few conserved residues in the signal peptide and across the rest of the sequence. Therefore, this hit was not able to identify this cluster and was not considered to be a part of this novel putative toxin-family.

#### Cluster 8: Novel neuropeptide toxins

The best BLASTp hit of this cluster was between “Andy_TR16535 c1_g1_i2” and orphan peptide AbOp-18 (AIX87708.1) from *Androctonus bicolor*. Another hit suggested similarity between this cluster and the venom neuropeptide-1 peptide found in in *Mesobuthus eupeus*. Because of the similarities, this cluster and the orphan peptide AbOp-18 were labelled as part of the same neuropeptide-family as and the venom neuropeptide-1 peptide found in in *Mesobuthus eupeus*. This family could be Buthidae specific.

#### Cluster 9: Novel toxins related to venom peptide meuTx23

The best BLASTp hit of this cluster was between “Gros_TR5239 c0_g1_i1” and venom toxin meuTx23 (AMX81480.1) from *Mesobuthus eupeus*. The transcripts in this cluster showed similarity to this BLASTp hit, especially in residues of the transcript outside the signal peptide. Therefore, the transcripts were labelled as putative venom toxins closely related to the venom toxin meuTx23.

#### Cluster 10: Novel putative secreted proteins

The best BLASTp hit of this cluster was between “Andy_TR15703 c0_g1_i1” and hypothetical secreted protein (ADY39511.1) from *Hottentotta judaicus*. This BLASTp hit was unable to identify this cluster due to low similarity, and because of the lack of a C-pattern this cluster is labelled as a novel putative secreted protein family.

#### Cluster 11: Novel putative secreted proteins

The best BLASTp hit had a very low similarity score between “Hot_TR11671 c0_g1_i1” and a putative RNA-binding protein (SCO66159.1) from *Plasmodium vivax*. Due to lack of high similarity BLAST hits, we could not assign a family or function to this cluster. Because of the lack of a C-pattern this cluster is labelled as a novel putative secreted protein family..

#### Cluster 12: Novel putative toxin-family 5

Transcripts of this cluster, especially transcript ”Hot_TR19149 c0_g1_i1”, are very similar to their best BLASTp hit which was uncharacterized protein (XP_023221782.1) from *Centruroides sculpturatus*. Since transcripts of this cluster have a typical conserved C-pattern, high telson specific expression and a signal peptide, together with the fact that the BLASTp hit was not previously linked to venom or toxins, this cluster and this uncharacterized protein were labelled as a novel putative toxin family.

#### Cluster 13: Novel alpha-potassium channel toxins

The best BLASTp hit of this cluster was between “Baby_TR13798_c1_g2_i2” and Potassium channel toxin alpha-KTx 4.5 (Q5G8B6.1) from *Tityus costatus*. Members of this cluster had a similar C-pattern to this BLASTp. Their signal peptides were quite dissimilar, but other residues across the peptide were conserved. Therefore, transcripts in this cluster were labelled as novel toxins in the alpha-KTx toxin-family.

#### Cluster 14: Novel putative secreted proteins

The best BLASTp hit of this cluster was between “Nebo_TR139281 c0_g3_i2” and hypothetical protein (AEX09189.1) from *Pandinurus cavimanus*. Both transcripts in this cluster showed similarity with this best BLASTp hit, which is a hypothetical protein, and thus unhelpful for further identification. Because of the lack of a C-pattern, transcripts in this cluster were labelled as novel hypothetical secreted proteins. Functional studies in the future could determine whether if these transcripts are toxins.

#### Cluster 15: Novel putative short toxin-family 6

The best BLASTp hit of this cluster was between “Iurus_TR23449 c0_g1_i1” and hypothetical protein (GAU10035.1) from *Trifolium subterraneum*. The best BLASTp hit of this cluster was unhelpful to identify this cluster because it was only a partial hit, but because of the presence of two cysteines in the two transcripts and their short lengths of 25 amino acids, this cluster was labelled as a novel putative short toxin-family. This peptide could potentially be promising for pharmaceutical use after functional studies.

#### Singlet 1: Novel putative secreted protein

This singlet did not have a BLASTp hit, and could not be identified. Furthermore, this singlet does not have a toxin-like structure. Therefore, this singlet was labelled as a novel putative secreted protein. Functional studies in the future could elaborate if this transcript is a NDBP toxin.

#### Singlet 2: Novel putative secreted protein

Much like singlet one this singlet did not have a BLASTp hit, and could not be identified. Furthermore, this singlet does not have a toxin-like structure. Therefore, this singlet was labelled as a novel putative secreted protein. Functional studies in the future could elaborate if this transcript is a NDBP toxin.

#### Singlet 3: Novel putative secreted protein

This singlet, “Iurus_TR30208 c0_g1_i1”, seems to be related to its best BLASTp hit, hypothetical protein (AEX09189.1) from *Pandinurus cavimanus*, but since this was a hypothetical protein this singlet was not further identified. Since this singlet also lacked a C-pattern, it was labelled as a novel putative secreted protein. Functional studies in the future could elaborate if this transcript is a NDBP toxin.

#### Singlet 4: Novel putative secreted protein

The best BLASTp hit of this singlet, orphan peptide AbOp-11 (AIX87714.1) from *Androctonus bicolor* was unhelpful for identifying this singlet, and due to the lack of a C-pattern this singlet was labelled as a novel putative secreted protein. Functional studies in the future could elaborate if this transcript is a NDBP toxin.

#### Singlet 5: Novel potassium channel toxin

The singlet “Iurus_TR10858 c0_g1_i1” had the same conserved C-pattern as the potassium channel toxin kappa-KTx (P0DJ41.1) from *Heterometrus laoticus*. However, both the signal peptide and residues across the peptide had almost no similarities. Therefore, it is possible that this is a new type of potassium channel toxin. Functional studies should follow to identify the target and structure of this putative toxin.

#### Singlet 6: Novel putative secreted protein

The best BLASTp hit of this singlet, venom peptide Htgkr2 (AOF40260.1) from *Hadogenes troglodytes* was unhelpful for identifying this singlet because of numerous dissimilarities. Furthermore, because of the lack of a C-pattern this singlet was labelled as a novel putative secreted protein. Functional studies in the future could elaborate if this transcript is a NDBP toxin..

#### Singlet 7: Novel putative toxin 1

This singlet found in *P. kraepelini* did not have any BLASTp hits. Therefore, it could not be further identified. Since this singlet does have a typical C-pattern, high telson specific expression and a signal peptide, this singlet is labelled as a novel putative toxin.

#### Singlet 8: Novel putative secreted protein

The best BLASTp hit of this singlet, “SH3 domain and tetratricopeptide repeat-containing protein” (XP_004574858.2) from *Maylandia zebra* was unhelpful with identifying this singlet because of numerous dissimilarities. Furthermore, because of the lack of a C-pattern this singlet was labelled as a novel putative secreted protein. Functional studies in the future could elaborate if this transcript is a NDBP toxin.

#### Singlet 9: Novel putative AMP

The best BLASTp hit of this singlet was between “Nebo_TR12128_c0_g1_i1” and putative antimicrobial peptide (AEX09192.1) from *Pandinurus cavimanus*. This singlet shows reasonable similarities to this putative AMP. Therefore, it is possible that this singlet belongs to the same AMP family or a related AMP family. This singlet was in the end labelled as a novel putative AMP.

Supplementary table 3: Origin of the samples with the coordinates of capture where available.

| **Scorpion family** | **Species name** | **Sample origin** | **Coordinates** |
| --- | --- | --- | --- |
| Buthidae | *Androctonus mauretanicus* | 20km WNW of Guelmin, Morocco | 29.068, -10.248 |
| Buthidae | *Babycurus gigas* | Pet trade |  |
| Buthidae | *Grosphus grandidieri* | Pet trade, Madagascar |  |
| Buthidae | *Hottentotta gentili* | 7km south of Tata, Morocco | 29.679, -7.981 |
| Iuridae | *Protoiurus kraepelini* | Fethiye, Muğla, Turkey |  |
| Diplocentridae | *Nebo hierichonticus* | Pet trade, Israel |  |
